# Continuation Versus Discontinuation of Buprenorphine in the Perioperative Setting: A Retrospective Study

**DOI:** 10.7759/cureus.23385

**Published:** 2022-03-22

**Authors:** Braden Schuster, Brooke Bell, Anthony Massoll, Seth White

**Affiliations:** 1 Anesthesiology and Perioperative Medicine, University of Alabama at Birmingham Hospital, Birmingham, USA; 2 Anesthesiology and Perioperative Medicine, University of Alabama at Birmingham Medical School, Birmingham, USA

**Keywords:** chronic pain, perioperative, postoperative pain, surgery, anesthesia, naloxone, subutex, suboxone, buprenorphine

## Abstract

Background

Buprenorphine use continues to grow for the management of opioid use disorder (OUD) and chronic pain management. In the face of this increase in use, perioperative buprenorphine management continues to have conflicting recommendations with no consensus on optimal management. We examined the effects of holding versus continuing perioperative buprenorphine in patients on chronic buprenorphine therapy to seek an answer to whether it should be continued or discontinued in the perioperative period.

Methods

Patients who were included in the study had surgery from 2011 to 2020 and had received buprenorphine within 30 days prior to their surgery, were admitted postoperatively for at least 48 hours, went to the postanesthesia care unit (PACU) immediately after surgery, and were successfully extubated. For these 275 patients, the included factors were age, gender, primary surgical service, anesthesia type, postoperative opioid use, preoperative regional block performed, and inpatient pain service (IPS) consultation. The analysis included differences between patients who had continued versus discontinued buprenorphine either preoperatively or postoperatively.

Results

A total of 275 patients were treated within 30 days of surgery with buprenorphine; of these, 147 (53.4%) patients continued buprenorphine, and 128 (46.6%) discontinued buprenorphine preoperatively. For patients who discontinued buprenorphine preoperatively, the mean days stopped before surgery was 3.5 days. Patients continuing buprenorphine preoperatively had a significantly lower postoperative opioid requirement. In addition, patients were significantly younger and more likely to be female and had fewer IPS consultations than those who discontinued buprenorphine. Buprenorphine was restarted postoperatively for 143 (52%) patients and held for 132 (48%) postoperatively.

Conclusions

The use of buprenorphine perioperatively was associated with significantly reduced oral morphine equivalent (OME) requirements postoperatively. Further research is needed to give definitive recommendations for whether to continue or discontinue buprenorphine prior to surgery.

## Introduction

Buprenorphine was first discovered in the 1960s, although not marketed in the United States until 1985 as an injectable schedule V narcotic analgesic [1]. Initially, its development was as an analgesic alternative to morphine to help avoid morphine’s known addictive properties and side effects. Today, with an estimated 50 million adults in the United States of America affected by chronic pain and increasing frequency of opioid use disorder (OUD), buprenorphine is being encountered more frequently in the perioperative setting.

Buprenorphine’s mechanism of action is via partial agonism at the µ-opioid receptor (MOR), antagonism at the κ-opioid receptor (KOR), and weak agonism at the δ-opioid receptor (DOR) [2-6]. This partial agonism is what led to its eventual use in opioid use disorder. The Drug Addiction Treatment Act of 2000 allowed, for the first time, schedule III, IV, and V medications to be prescribed by a qualified physician outside of a federally approved opioid treatment program [1]. This included a caveat that the medications must have FDA approval for the treatment of opioid use disorder. The FDA then approved buprenorphine and buprenorphine-naloxone combination in 2002 for use in managing opioid dependence [3,6]. This led to a large increase in the use of buprenorphine. The Substance Abuse and Mental Health Services Administration (SAMHSA) national survey showed that in non-opioid treatment programs the number of patients on buprenorphine increased from 1,670 in 2004 to 54,488 in 2015. Similar percentage increases were also seen in opioid treatment programs [7].

In addition to buprenorphine’s partial MOR agonism, there are several other characteristics that are ideal for the treatment of OUD. Buprenorphine has a high potency, approximately 30 times more potent than morphine, while also having a slower development of tolerance [2,3,5]. When comparing opioids, only fentanyl and its derivatives have a higher relative potency than buprenorphine. Its high-affinity ligand binding and slow receptor dissociation cause buprenorphine to have a high occupancy of MORs [2-6]. Even at low doses, this leads to a competitive displacement of traditional full opioid agonists off opioid receptors. At sublingual doses of 16 mg, MOR availability is reduced by 79%-95% [2,8]. This displacement can last for several days as buprenorphine has a long and variable half-life, ranging from 16.4 hours to 70 hours [3,4]. Due to its partial agonism, there is an analgesic ceiling effect in which further increases in dosing will not produce any greater analgesic effect [4,6]. Finally, when buprenorphine is combined with naloxone, there is a decreased risk for abuse. If intravenously injected, naloxone will counteract buprenorphine’s effect, but if taken properly, it will have no effect due to its poor sublingual bioavailability [3,5].

While these characteristics are beneficial for its intended use in OUD, it complicates the treatment of perioperative pain. In addition, there is concern that buprenorphine will inhibit the analgesic effects of full opioid agonists that are inevitably used perioperatively. Inhibition then leads to, among other things, uncontrolled postoperative pain, increase length of stay (LOS), and patient dissatisfaction [2,3]. Over the years, various opinions have been formed on the best way to manage buprenorphine prior to surgery. Unfortunately, there is currently no high-level evidence supporting either the continuation or discontinuation of buprenorphine perioperatively [2,5,6].

## Materials and methods

**Figure 1:**
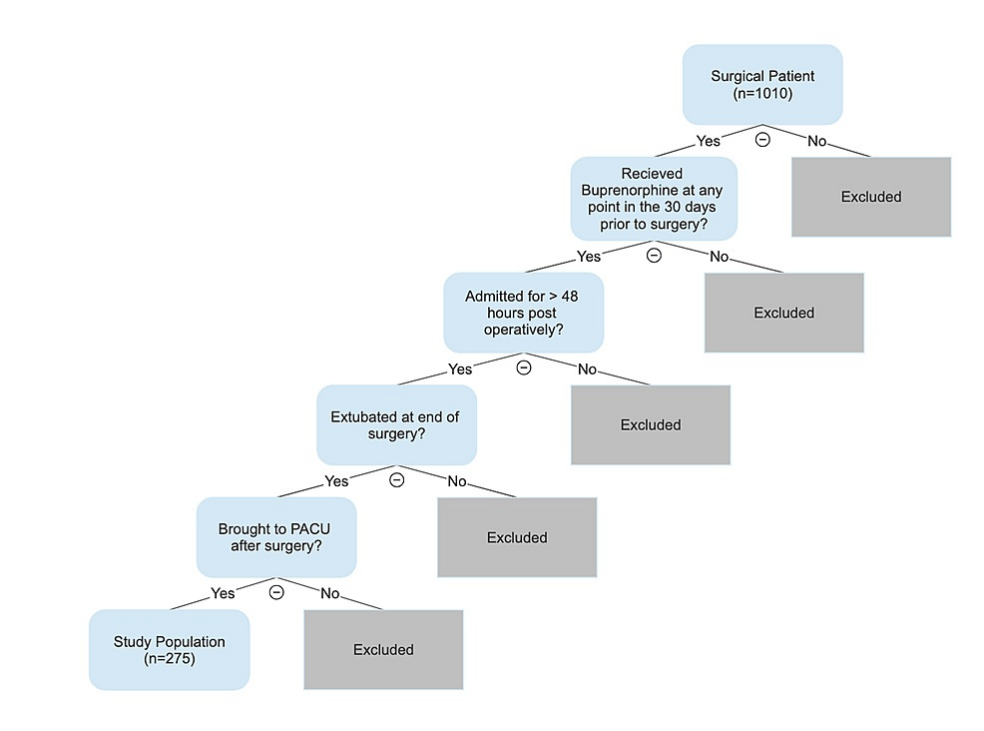
Study population with exclusion criteria

The University of Alabama at Birmingham’s Institutional Review Board approved the study under an exempt status. The study patients for this case series were then selected via a query of the University of Alabama at Birmingham electronic medical record (EMR) system with the assistance of the hospital anesthesia information technology department. A search was performed from January 1, 2011, to December 31, 2020, of all surgical patients with a previously documented history of buprenorphine use. This resulted in 1,010 possible study patients, which was further narrowed down using the following exclusion criteria: most recent buprenorphine use greater than 30 days prior to surgery, LOS less than 48 hours, patients who bypassed the postanesthesia care unit (PACU), and patients who remained intubated postoperatively (Figure 1). The final study population included 275 patients whose medical records were then individually reviewed by an author to ensure they met the criteria.

Further data was then obtained for the final study population from a review of the EMR system. This included the patients’ age, gender, surgical service who performed the operation, anesthesia type (general, monitored anesthesia care, neuraxial, and regional), continuation versus discontinuation of buprenorphine prior to surgery, preoperative regional block performed (none, single-shot injection, catheter placement, or single-shot injection and catheter placement), and inpatient pain service (IPS) consultation. For patients who held their buprenorphine preoperatively, the number of days held was noted (1-3 days, 4-7 days, and ≥8 days). Postoperative opioid use was evaluated and recorded in PACU, PACU discharge to 24 hours postoperatively, and 24-48 hours postoperatively. All opioid full agonists used during this time were converted to oral morphine milligram equivalents (MMEs) to allow direct comparison.

SAS version 9.4 (SAS Institute Inc., Cary, NC, USA) was used to conduct all statistical analyses. Data are expressed as means and standard errors (for continuous variables) or numbers and percentages (for categorical variables). Two-sample t-tests and chi-square tests were used to compare the two groups. Normality for continuous outcomes was assessed using probability plots and the Shapiro-Wilk test for normality. For any outcomes where normality could not be reasonably assumed, the Wilcoxon rank-sum test was used in place of t-test. All tests of statistical significance were two-sided and used a significance level of 5%.

## Results

Data were available for 275 patients. For 147 (53.4%) patients, buprenorphine was continued preoperatively; for the remaining 128 (46.6%) patients, buprenorphine was discontinued preoperatively. For patients where buprenorphine was discontinued preoperatively, the mean (standard error) days stopped before surgery was 3.5 (0.4) days. The demographic and clinical characteristics of the sample by preoperative buprenorphine are shown in Table 1. Patients continuing buprenorphine preoperatively were significantly younger and more likely to be female and had fewer IPS consultations than those who discontinued buprenorphine. Patients continuing buprenorphine also had significantly lower opioid requirements than those who discontinued buprenorphine. There was no significant difference in hospital length of stay between the two groups (Table 2).

**Table 1:** Demographic and clinical characteristics by preoperative buprenorphine *P-value from two-sample t-test (age) or chi-square test (gender, regional block, anesthesia type, and IPS consultation)

Characteristic	Continued (n = 147)	Discontinued (n = 128)	P*
Age, mean (SE)	38.68 (1.11)	44.64 (1.26)	<0.001
Gender, n (%)			<0.001
Female	116 (78.91%)	68 (53.13%)	
Male	31 (21.09%)	60 (46.88%)	
Days discontinued, n (%)			
1–3		98 (76.56%)	
4–7		15 (11.72%)	
8+		15 (11.72%)	
Preoperative regional block, n (%)			0.050
Catheter	1 (0.68%)	6 (4.69%)	
SS	16 (10.88%)	14 (10.94%)	
Catheter and SS	4 (2.72%)	0 (0%)	
None	126 (85.71%)	108 (84.38%)	
Anesthesia type, n (%)			<0.001
General	55 (37.41%)	105 (82.03%)	
MAC	5 (3.40%)	6 (4.69%)	
Neuraxial	86 (58.50%)	17 (13.28%)	
Regional	1 (0.68%)	0 (0%)	
IPS consulted, n (%)			0.039
No	139 (94.56%)	112 (87.50%)	
Yes	8 (5.447%)	16 (12.50%)	

**Table 2:** Oral morphine milligram equivalent requirements and length of stay by preoperative buprenorphine *P-value from two-sample t-test

Outcome	Continued (n = 147)	Discontinued (n = 128)	P*
Oral MME, mean (SE)			
PACU	63.89 (14.86)	123.95 (20.59)	0.017
0–24 hours	100.22 (23.84)	220.96 (28.32)	0.001
24–48 hours	66.40 (20.90)	190.95 (27.76)	<0.001
Hospital length of stay, mean (SE)	6.11 (0.65)	7.44 (0.89)	0.222

Buprenorphine was restarted postoperatively for 143 (52%) patients and held for 132 (48%) patients. Postoperative buprenorphine was significantly associated with preoperative buprenorphine (p < 0.001): 78.3% of those who continued buprenorphine preoperatively restarted following surgery, and 73.5% of those who discontinued preoperatively had buprenorphine held after surgery.

For patients where buprenorphine was restarted, the mean (SE) days before restarting was 0.3 (0.1) days. The demographic and clinical characteristics of the sample by postoperative buprenorphine are shown in Table 3. Patients restarting buprenorphine were significantly younger and more likely to be female and had fewer inpatient pain service (IPS) consultations than those who did not restart buprenorphine. Patients restarting buprenorphine also had significantly lower opioid requirements than those who did not restart buprenorphine but did not significantly differ in hospital length of stay (Table 4).

**Table 3:** Demographic and clinical characteristics by postoperative buprenorphine *P-value from two-sample t-test (age) or chi-square test (gender, regional block, anesthesia type, and IPS consultation)

Characteristic	Held (n = 132)	Restarted (n = 143)	P*
Age, mean (SE)	48.89 (1.21)	34.59 (0.87)	<0.001
Gender, n (%)			<0.001
Female	63 (47.73%)	121 (84.62%)	
Male	69 (52.27%)	22 (15.38%)	
Preoperative regional block, n (%)			0.251
Catheter	5 (3.79%)	2 (1.40%)	
SS	17 (12.88%)	13 (9.09%)	
Catheter and SS	3 (2.27%)	1 (0.70%)	
None	107 (81.06%)	127 (88.81%)	
Anesthesia type, n (%)			<0.001
General	120 (90.91%)	40 (27.97%)	
MAC	6 (4.55%)	5 (3.50%)	
Neuraxial	5 (3.79%)	98 (68.53%)	
Regional	1 (0.76%)		
IPS consulted, n (%)			<0.001
No	112 (84.85%)	139 (97.20%)	
Yes	20 (15.15%)	4 (2.80%)	

**Table 4:** Opioid requirements and length of stay by postoperative buprenorphine *P-value from two-sample t-test

Outcome	Held (n = 132)	Restarted (n = 143)	P*
Opioid requirements, mean (SE)			
PACU	148.60 (23.40)	39.46 (8.84)	<0.001
0–24 hours	239.90 (29.02)	79.36 (22.08)	<0.001
24–48 hours	218.49 (33.41)	37.50 (8.34)	<0.001
Hospital length of stay, mean (SE)	6.43 (0.57)	6.97 (0.89)	0.617

## Discussion

For patients on buprenorphine therapy in the perioperative period, most commonly, you can divide the treatment plan into three options: continue buprenorphine throughout the perioperative period, discontinue buprenorphine for a period prior to surgery, or increase the buprenorphine dose preoperatively to a maximum dose of 32 mg/day [3,4,9]. Unfortunately, the present study does not include any patients whose preoperative buprenorphine dose was changed; thus, our main concern was directly comparing patients who had their buprenorphine continued versus discontinued in the perioperative period.

Recommendations for the management of buprenorphine in the perioperative period have varied over time. A common approach described by Anderson et al. [2] and Jonan et al. [3] is to first risk stratify the surgery into the expected level of postoperative pain (minimal to no pain versus moderate to severe pain) [2] or expected postoperative opioid requirements (low versus high) [3]. This is then further divided to look at elective surgery versus emergency surgery. In elective surgery, if minimal to no pain or low opioid requirement is expected, patients who are still actively taking buprenorphine should continue this perioperatively. In surgeries where moderate to severe pain or high opioid requirement is expected, they recommended discontinuing buprenorphine prior to surgery and postponing surgical intervention if the patient had not yet stopped buprenorphine therapy. For urgent and emergent surgery, if minimal to no pain or low opioid requirement is expected, it is recommended for patients who are still actively taking buprenorphine to continue this perioperatively. For emergent surgeries with moderate to severe pain or if high opioid requirements are expected, buprenorphine should be discontinued and not restarted in the immediate postoperative period.

These recommendations were based on concerns with inability to achieve adequate pain control due to buprenorphine’s pharmacodynamic and pharmacokinetic properties. Although described previously, buprenorphine has an analgesic ceiling effect and high receptor binding affinity for the μ-receptor that causes supplemental full μ-agonist opioids to be less efficacious [2,3,6]. More recently, there has been a trend to increase circumstances in which buprenorphine is continued perioperatively, especially in OUD patients [10]. Lembke et al. [11] published an editorial in which their recommendations were to assess the patient’s daily buprenorphine dose. If the patient’s buprenorphine dose was greater than 12 mg, the recommendation was to reduce the dose to 12 mg two to three days prior to surgery and restart their normal dose postoperatively. If the patient’s buprenorphine dose was 12 mg or less, they should continue their home regimen throughout the perioperative period. This was based on the belief that buprenorphine’s MOR blockade is dose-dependent [5,8], and risks are associated with the discontinuation of therapy in OUD. These risks with buprenorphine discontinuation include opioid withdrawal, relapse, and exacerbation of chronic pain [5].

The Perioperative Pain and Addiction Interdisciplinary Network (PAIN) published a clinical practice advisory with recommendations to continue buprenorphine therapy in most circumstances in the perioperative period [6]. Further recommendations include initiating full μ-agonists if analgesia is inadequate while still continuing the patient’s buprenorphine therapy. This is most consistent with the results of the present study, although the clinical practice advisory is based largely on class 4 and 5 evidence.

Our findings show that patients had a significantly decreased postoperative opioid requirement if they continued buprenorphine preoperatively or were restarted on buprenorphine postoperatively. This was consistent across all postoperative time periods: PACU, PACU discharge to 24 hours postoperatively, and 24-48 hours postoperatively. Of the patients who continued buprenorphine preoperatively, 78.3% had their buprenorphine restarted in the postoperative period. Regarding the type of anesthesia administered, patients who continued buprenorphine had a significantly higher percentage of neuraxial anesthesia. Obtaining a preoperative regional block had no significant effect on whether buprenorphine was held or continued postoperatively.

Multiple limitations exist in this study. First, this is a retrospective design. Being a retrospective study, it is at higher risk for confounding. Our data shows that for the groups who had their buprenorphine continued preoperatively and/or restarted postoperatively, patients tended to be younger and female and undergo neuraxial anesthesia. This could potentially be due to having a higher number of obstetrics or gynecological surgical procedures in this group. Second, we were unable to risk stratify patients’ surgical procedures into the level of expected postoperative opioid requirements. Because of this, we are unable to comment as to the reason some patients may have had their buprenorphine held. Third, intraoperative opioid use was not taken into consideration. While this may have some effect on initial PACU opioid dosing, postoperative opioid dosing is guided using a pain rating scale. The IPS was consulted on a significantly higher percentage of patients who had their buprenorphine held postoperatively. This may be solely due to the primary team holding buprenorphine and automatically triggering an IPS consult, but the exact cause is unknown.

Managing perioperative pain for patients on chronic buprenorphine therapy is a delicate situation. There are concerns that uncontrolled pain postoperatively in patients on chronic buprenorphine therapy has an increased risk of postoperative opioid abuse relapse [3]. This risk is weighed against the possibility that holding buprenorphine postoperatively may cause an exacerbation of the original underlying pain [6] or an increased rate of illicit drug use if not restarted appropriately [5,12]. Utilizing multimodal analgesia has been repeatedly shown to improve postoperative pain [13] and is essential for these patients. Regardless, these patients may have high opioid requirements whether they continue or discontinue buprenorphine [4].

## Conclusions

The continuation or resumption of buprenorphine postoperatively significantly reduced oral MME requirements in our study population while having no prolonging effect on hospital length of stay. In addition, patients maintained on postoperative buprenorphine required fewer IPS consultations, suggesting that they had superior pain control compared with patients not maintained on buprenorphine postoperatively. While there are significant limitations to the study, our data suggest that the concerns surrounding pain control and extended length of hospital stay are not supported. The question of which patients will and will not benefit from continuation of buprenorphine in the perioperative setting according to varying levels of pain associated with surgery types has not yet been answered. Buprenorphine’s effects on patient satisfaction and postoperative pain control also have not been definitively answered. A prospective study may be warranted for more definitive answers.
